# Prospective evaluation of the relevance of Epstein–Barr virus antibodies for early detection of nasopharyngeal carcinoma in Chinese adults

**DOI:** 10.1093/ije/dyae098

**Published:** 2024-07-15

**Authors:** Ling Yang, Christiana Kartsonaki, Julia Simon, Pang Yao, Yu Guo, Jun Lv, Robin G Walters, Yiping Chen, Hannah Fry, Daniel Avery, Canqing Yu, Jianrong Jin, Alexander J Mentzer, Naomi Allen, Julia Butt, Michael Hill, Liming Li, Iona Y Millwood, Tim Waterboer, Zhengming Chen

**Affiliations:** Clinical Trial Service Unit & Epidemiological Studies Unit (CTSU), Nuffield Department of Population Health, University of Oxford, Oxford, UK; Clinical Trial Service Unit & Epidemiological Studies Unit (CTSU), Nuffield Department of Population Health, University of Oxford, Oxford, UK; Infections and Cancer Epidemiology Division, German Cancer Research Center (DKFZ), Heidelberg, Germany; Clinical Trial Service Unit & Epidemiological Studies Unit (CTSU), Nuffield Department of Population Health, University of Oxford, Oxford, UK; National Center for Cardiovascular Diseases, Fuwai Hospital Chinese Academy of Medical Sciences, Beijing, China; Department of Epidemiology and Biostatistics, School of Public Health, Peking University Health Science Centre, Beijing, China; Peking University Center for Public Health and Epidemic Preparedness & Response, Peking University, Beijing, China; Clinical Trial Service Unit & Epidemiological Studies Unit (CTSU), Nuffield Department of Population Health, University of Oxford, Oxford, UK; Clinical Trial Service Unit & Epidemiological Studies Unit (CTSU), Nuffield Department of Population Health, University of Oxford, Oxford, UK; Clinical Trial Service Unit & Epidemiological Studies Unit (CTSU), Nuffield Department of Population Health, University of Oxford, Oxford, UK; Clinical Trial Service Unit & Epidemiological Studies Unit (CTSU), Nuffield Department of Population Health, University of Oxford, Oxford, UK; Department of Epidemiology and Biostatistics, School of Public Health, Peking University Health Science Centre, Beijing, China; Peking University Center for Public Health and Epidemic Preparedness & Response, Peking University, Beijing, China; NCD Department, Wuzhong CDC, Suzhou, China; The Wellcome Centre for Human Genetics, University of Oxford, Oxford, UK; Clinical Trial Service Unit & Epidemiological Studies Unit (CTSU), Nuffield Department of Population Health, University of Oxford, Oxford, UK; Infections and Cancer Epidemiology Division, German Cancer Research Center (DKFZ), Heidelberg, Germany; Clinical Trial Service Unit & Epidemiological Studies Unit (CTSU), Nuffield Department of Population Health, University of Oxford, Oxford, UK; Department of Epidemiology and Biostatistics, School of Public Health, Peking University Health Science Centre, Beijing, China; Peking University Center for Public Health and Epidemic Preparedness & Response, Peking University, Beijing, China; Clinical Trial Service Unit & Epidemiological Studies Unit (CTSU), Nuffield Department of Population Health, University of Oxford, Oxford, UK; Infections and Cancer Epidemiology Division, German Cancer Research Center (DKFZ), Heidelberg, Germany; Clinical Trial Service Unit & Epidemiological Studies Unit (CTSU), Nuffield Department of Population Health, University of Oxford, Oxford, UK

**Keywords:** Nasopharyngeal cancer, Epstein–Barr Virus (EBV), multiplex serology assay, case-cohort study, early detection, China

## Abstract

**Background:**

Epstein–Barr virus (EBV) is a major cause of nasopharyngeal carcinoma (NPC) and measurement of different EBV antibodies in blood may improve early detection of NPC. Prospective studies can help assess the roles of different EBV antibodies in predicting NPC risk over time.

**Methods:**

A case-cohort study within the prospective China Kadoorie Biobank of 512 715 adults from 10 (including two NPC endemic) areas included 295 incident NPC cases and 745 subcohort participants. A multiplex serology assay was used to quantify IgA and IgG antibodies against 16 EBV antigens in stored baseline plasma samples. Cox regression was used to estimate adjusted hazard ratios (HRs) for NPC and C-statistics to assess the discriminatory ability of EBV-markers, including two previously identified EBV-marker combinations, for predicting NPC.

**Results:**

Sero-positivity for 15 out of 16 EBV-markers was significantly associated with higher NPC risk. Both IgA and IgG antibodies against the same three EBV-markers showed the most extreme HRs, i.e. BGLF2 (IgA: 124.2 (95% CI: 63.3–243.9); IgG: 8.6 (5.5–13.5); LF2: [67.8 (30.0–153.1), 10.9 (7.2–16.4)]); and BFRF1: 26.1 (10.1–67.5), 6.1 (2.7–13.6). Use of a two-marker (i.e. LF2/BGLF2 IgG) and a four-marker (i.e. LF2/BGLF2 IgG and LF2/EA-D IgA) combinations yielded C-statistics of 0.85 and 0.84, respectively, which persisted for at least 5 years after sample collection in both endemic and non-endemic areas.

**Conclusions:**

In Chinese adults, plasma EBV markers strongly predict NPC occurrence many years before clinical diagnosis. LF2 and BGLF2 IgG could identify NPC high-risk individuals to improve NPC early detection in community and clinical settings.

Key Messages

- Measurement of multiple EBV antibodies, both IgA and IgG, in blood may improve early detection of NPC, but no prospective evidence is available on the role of different EBV antibodies in predicting NPC risk over time.
- This case-cohort study of participants from both endemic and non-endemic NPC areas in China showed that sero-positivity for 15 EBV antigens was significantly associated with a higher risk of NPC.
- Use of only two IgG-based EBV-markers can reliably predict the occurrence of NPC for at least 5 years before hospital diagnosis in both NPC endemic and non-endemic areas.

## Introduction

Although relatively rare globally, nasopharyngeal carcinoma (NPC) has a distinct geographical and ethnic distribution, with almost half of new cases worldwide occurring in China.^1^ Within China, there is also a large regional variation in NPC incidence, with the rates in certain southern regions of China being >25 times as high as in other parts of China.^2^ Given non-specific clinical symptoms, diagnosis of NPC is often delayed until it is at an advanced stage when 5-year survival is typically <50%, as opposed to >95% when diagnosed at an early stage.^1^^,^^3^ Epstein–Barr virus (EBV) infection has been recognized as a necessary step in the development of NPC.^4^^,^^5^ Although EBV is a ubiquitous pathogen with 95% of the world population infected, NPC develops in only a small fraction of infected individuals; thus identification of NPC-specific EBV biomarkers is particularly important to aid NPC early detection.^6^^,^^7^

Several studies have demonstrated that detection of certain EBV markers appeared to reliably predict NPC diagnosis,^8–12^ leading to the development of NPC early detection serology based chiefly on immunofluorescence and enzyme-lined immunosorbent assays (ELISA). Such assays historically test IgA antibody levels against the viral capsid antigen (VCA), EBV nuclear antigen 1 (EBNA1) and/or early antigen (EA), but often reported relatively low positive predictive value.^12–16^ Higher early diagnostic rate of NPC in the screen arm (46%) compared with the control arm (21%) was demonstrated in a cluster randomized controlled screening trial in Southern China.^9^ More than three historically measured EBV markers relate to multi-protein complexes^17^ in which each protein and each peptide sequence within the protein could serve as a reasonable sero-marker target in NPC screening/early detection approach. Furthermore, compared with IgA, IgG is a more abundant systemic antibody and reflects longer-term exposure to lytic viral activity.^18^ We investigated the possibility of improving of NPC early detection by incorporating both IgA and IgG antibodies against NPC-specific EBV markers.^19–21^

By using a whole-proteome microarray measuring 199-marker anti-EBV peptide sequences from 86 EBV proteins, 14 NPC-specific EBV markers have recently been identified, which showed ∼10% improvement in the accuracy rate for predicting NPC onset compared with VCA and EBNA1 IgA alone, which have been used in conventional NPC screening programmes.^22^ To facilitate the large-scale testing, the microarray technique has been further adapted into a Luminex bead-based serology assay. This serology assay validated the above NPC-specific EBV-markers, expressed as glutathione-S-transferase (GST) fusion proteins, and has been further evaluated in populations with intermediate (Taiwan)^23^ and low (UK)^24^ risk of NPC in clinical case-control settings. Besides the repeated high predictive accuracy for both overall and early-stage NPC diagnosis using the full-marker panel, two parsimonious panels (one involving four EBV IgA and IgG markers and another with only two IgG markers) were further identified, which showed 99.2% and 98.4% accuracy in detecting clinically diagnosed NPC, respectively.^23^ To help inform the NPC early detection in general populations, external population-based prospective data are required to further validate the role of these serological biomarkers in predicting future risk of NPC in general populations.

We present relevant findings from a case-cohort study within the prospective China Kadoorie Biobank (CKB). The aims of the present study were to: (i) investigate prospective associations of sero-positivity of individual IgA and IgG antibodies against 16 EBV antigens with risks of NPC; and (ii) assess the prediction value of two previously identified parsimonious EBV-marker combinations for NPC, overall and separately in NPC endemic and non-endemic areas.

## Methods

### Study population

Details on the CKB design, study population and survey methods have been reported previously.^25^ Briefly, CKB included 512 715 Chinese adults (59% women) aged 30–79 years, who were recruited from 10 geographically defined regions in China, including two located in NPC high-risk regions in Southern China (Supplementary Figure S1, available as Supplementary data at *IJE* online) during 2004–08.^25^ Prior international, national and regional ethical approvals were obtained, and all participants provided written informed consent.

At baseline, participants completed an interviewer-administered laptop-based questionnaire on sociodemographic and lifestyle factors (e.g. smoking, alcohol drinking and diet), and personal and family medical history. Moreover, a range of physical measurements was undertaken by trained technicians, along with collection of a 10-ml non-fasting blood for long-term storage. Periodical resurveys were conducted every 4–5 years among a random subset (5%) of surviving participants.

### Follow-up for incident cancer and other health outcomes

The vital status and health outcomes of each participant were monitored through electronic linkage with morbidity and mortality registries, as well as with the national health insurance system which has almost universal coverage in the study areas.^25^ All reported disease events were coded using ICD-10 by trained staff who were blinded to baseline information.

By 1 January 2017, 44 037 (8.6%) participants had died, 27 903 (5.4%) had developed cancer and 4781 (0.9%) were lost to follow-up.

### Case-cohort study design

The present case-cohort study included 295 incident NPC cases (ICD-10: C11) recorded up to 1 January 2017, and 745 randomly selected subcohort individuals. Both NPC cases and subcohort individuals had no prior history of any cancer and were alive 2 years after entering the study (Supplementary Figure S2, available as Supplementary data at *IJE* online).

### EBV serology assay

A multiplex serology assay^26^ was used to detect both IgA and IgG antibodies against 16 EBV antigens plus three human polyomaviruses antigens (as negative controls) in the baseline plasma samples. Details about sample preparation and antibodies selection are shown in Supplementary Materials (available as Supplementary data at *IJE* online). Median fluorescence intensity (MFI) values were generated from the multiplex serology assay for individual markers measured. The cancer-specific MFI cut-offs (Supplementary Table S1, available as Supplementary data at *IJE* online), that been used to define ‘sero-positivity’ for individual antibodies, were defined by German Cancer Research Center (DKFZ) based on previous studies^23^ in conjunction with percentile plots for antigens from general population samples.

### Statistical analysis

Age-specific cumulative incidence and direct CKB cohort age-sex-standardized incidence rates of NPC were calculated.

Cox proportional hazards models, fitted using the Prentice estimator, were used to estimate hazard ratios (HRs) for NPC associated with each EBV marker as a binary variable based on the pre-specified cut-off value, and with the two previously identified parsimonious marker combinations.^23^ The analyses were done in the overall regions and separately in endemic and non-endemic areas, adjusting for age (years), sex (male/female), study area (10 areas) and education (six categories). Time in study (i.e. time from entry into the study to cancer diagnosis, loss to follow-up, death due to other causes or 1 January 2017, whichever occurred first) was used as the time scale in the model, with the proportional hazards assumption assessed using Schoenfeld residuals.^27^

C-statistics^28^ were used in this case-cohort study to assess the discriminatory ability of each of the two previously identified parsimonious EBV-marker combinations for predicting risk of NPC,^23^ with two models fitted for each combination. The first model included each binary EBV-marker as an explanatory variable, and the second model used the published linear predictor of the original validated algorithms for calculating the adjusted HRs for NPC per unit increase in logit(*p*). For the four EBV-marker combination, logit(*p*) = –5.0015 + 3.4328 × BMRF1 IgA + 2.6795 × LF2 IgA + 3.5153 × BGLF2 IgG + 2.4180 × LF2 IgG), and for the two EBV-marker combination, logit(*p*) = –4.4302 + 4.4580 × BGLF2 IgG + 3.9562 × LF2 IgG).^23^

To further assess whether HRs varied with time since blood sample collection, models including an interaction with piecewise constant follow-up time were fitted. Models were also fitted with additional adjustment sequentially for smoking, alcohol drinking and family history of cancer. Further analyses were also conducted in subgroups defined by the above risk factors, in NPC endemic and non-endemic areas, and by time since blood sample collection.

Statistical analysis was done using R version 4.1.3.

## Results

**Figure 1.**
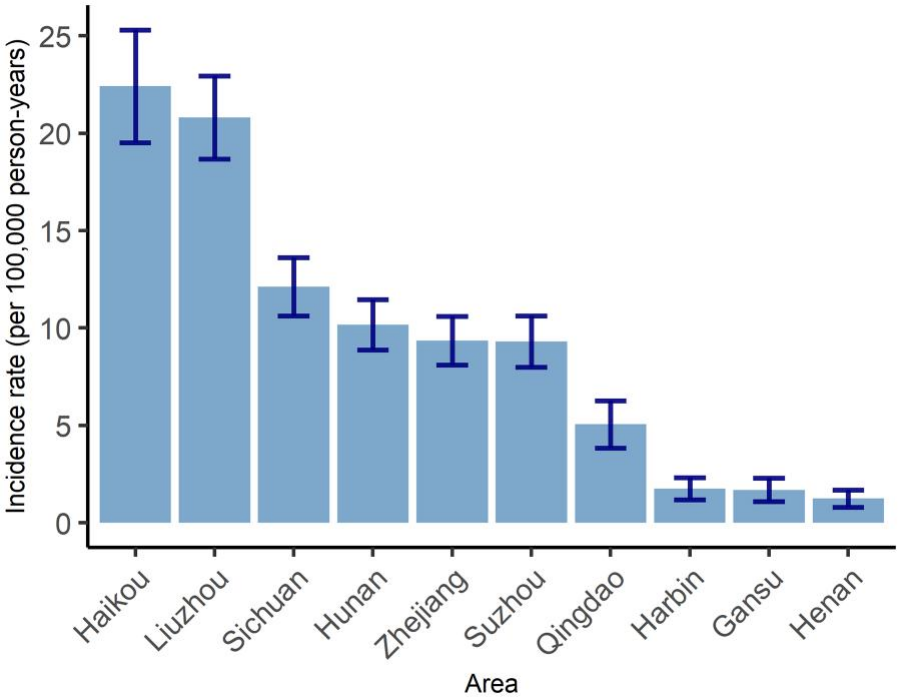
Age- and sex-standardized incidence rate of nasopharyngeal cancer in the 10 study areas. The rates were standardized to the age- (in groups 30–49, 50–59 and 60–79) and sex-distribution of the whole China Kadoorie Biobank (CKB) cohort. The vertical bars extend to ± standard error of the incidence rate

Overall the incidence rate of NPC in the whole CKB cohort was 8.6 per 100 000 person-years (*N* = 370), with the rates higher in men than in women (12.8 vs 5.8 per 100 000) and higher at older than younger ages in both sexes (Supplementary Figure S3, available as Supplementary data at *IJE* online). Two study areas located in southern China had significantly higher incidence rates of NPC (22.4 and 20.8 per 100 000 in Haikou and Liuzhou, respectively) than other areas where NPC rates were typically around 10 per 100 000 or below (Figure 1). These two areas are henceforth referred to as NPC ‘endemic areas’.

Compared with the subcohort participants, irrespective of endemic status, those with incident NPC were less likely to consume fresh fruits but more likely to be men, to smoke and drink alcohol, and have lower body mass index (BMI) and a family history of cancer at baseline. However, compared with those from non-endemic areas, participants in endemic areas, both involving urban cities, were less likely to smoke and drink alcohol, more likely to consume meat and fish but less likely to consume preserved vegetables (Table 1).

**Table 1.** Selected baseline characteristics of NPC cases and subcohort participants, overall and by endemic status of study area

	Endemic area		Non-endemic area			Overall		
	NPC cases (*N* = 106)	Subcohort (*N* = 122)	NPC cases (*N *= 189)	Subcohort (*N* = 623)	*P* ^a^	NPC cases (*N* = 295)	Subcohort (*N* = 745)	*P* ^b^
Age at study entry (years), mean (SD)	54.0 (9.9)	52.9 (11.0)	53.1 (10.2)	51.3 (10.3)	0.018	53.4 (10.1)	51.5 (10.4)	0.008
Women, %	49.1	63.9	38.6	60.5	<0.001	42.4	61.1	<0.001
Age at diagnosis (years), mean (SD)	60.0 (10.2)	63.1 (11.0)	59.0 (10.1)	61.5 (10.2)	0.002	59.4 (10.2)	61.8 (10.3)	0.001
Ever regular smoking, %	36.8	24.6	51.9	30.5	<0.001	46.4	29.5	<0.001
Ever regular alcohol drinking, %	10.4	7.4	31.2	17.0	<0.001	23.7	15.4	0.002
Formal education ≥6 years, %	70.8	73.0	38.1	48.8	<0.001	52.8	52.8	0.435
Household income ≥20000 yuan, %	39.6	39.3	43.4	45.4	0.491	42.0	44.4	0.527
Had a household fridge, %	72.6	77.0	39.7	54.1	<0.001	51.5	57.9	0.074
Overweight/obese (BMI ≥ 25 kg/m^2^), %	24.5	36.9	26.5	36.3	0.013	25.8	36.4	0.001
History of COPD, %	1.9	1.6	2.6	3.0	0.781	2.4	2.8	0.851
History of diabetes, %	9.4	4.1	4.2	4.8	0.190	6.1	4.7	0.440
History of TB infection, %	1.9	1.6	1.1	0.6	0.526	1.4	0.8	0.640
Family history of cancer, %	14.2	10.7	22.2	19.9	0.032	19.3	18.4	0.795
Daily consumption, %								
Meat	62.3	63.9	25.4	25.5	<0.001	38.6	31.8	0.043
Fish	9.4	11.5	1.6	1.8	<0.001	4.4	3.4	0.528
Preserved vegetables	1.9	0.8	24.3	20.2	<0.001	16.3	17.0	0.834
Fresh fruit	19.8	24.6	11.1	20.4	0.012	14.2	21.1	0.015

NPC, nasopharyngeal cancer; SD, standard deviation; BMI, body mass index; COPD, chronic obstructive pulmonary disease; TB, tuberculosis.

a *P*-value for testing endemic area cases, endemic area subcohort, non-endemic area cases, and non-endemic area subcohort means or proportions are equal (vs there is a difference between any two groups).

b *P*-value for testing that cases and subcohort means or proportions are equal.

The distribution of MFI values of IgA and IgG antibodies against each antigen measured with the relevant cut-off value among the 745 subcohort plasma is shown in Supplementary Figure S4 (available as Supplementary data at *IJE* online). No significant correlations were observed between most IgA and IgG antibodies against the same or different antigens (Supplementary Figure S5, available as Supplementary data at *IJE* online).

**Figure 2.**
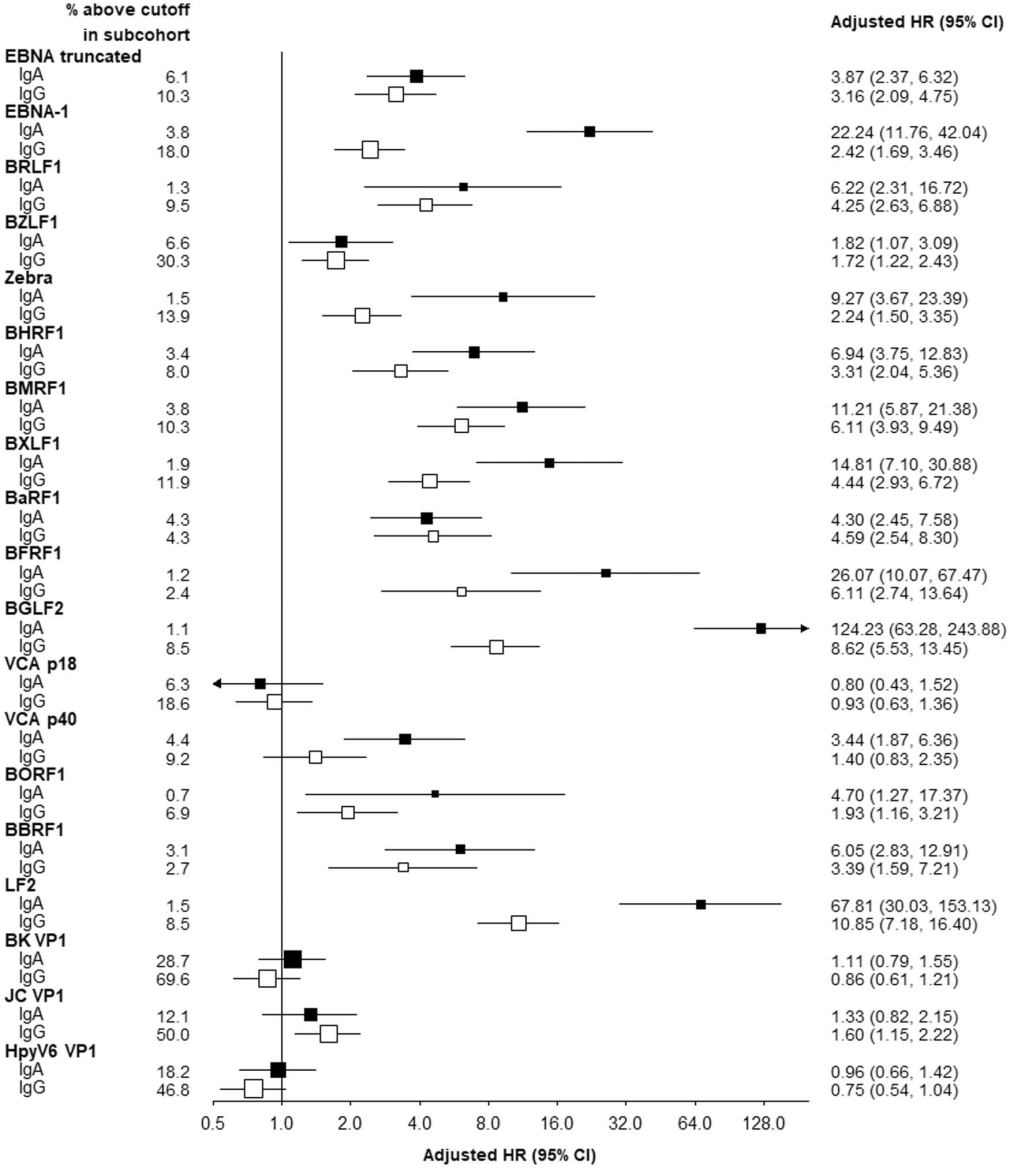
Adjusted hazard ratios for nasopharyngeal carcinoma associated with sero-positivity of IgA and IgG antibodies against each Epstein–Barr virus antigen. Models were adjusted for age (continuous), sex, area (10 areas) and education (six groups). The boxes indicate hazard ratios (HRs) for different Epstein–Barr virus markers (solid for IgA and open for IgG antibodies), with the area of each box inversely proportional to the variance of the logHR. The horizontal lines represent 95% confidence intervals (CIs)

Compared with those who were negative for specific EBV markers, with the exception of general infection marker VCAp18, individuals who were sero-positive for any of the other 15 anti-EBV markers had significantly higher risks of NPC. Due to the general low IgA-EBV reactivity in healthy carriers,^18^ higher correlations were observed among most IgG antibodies than IgA antibodies, and sero-positivity of IgA antibodies was associated with somewhat greater HRs compared with sero-positivity for IgG antibodies against same EBV antigens. For IgA antibodies, the three showing the most extreme HRs were BGLF2 (HR = 124.2, 95% CI: 63.3–243.9), LF2 (67.8, 30.0–153.1) and BFRF1 (26.1, 10.1–67.5). For IgG antibodies, the same three markers showed the highest risk estimates, with the corresponding HRs of 8.6 (5.5–13.5), 10.9 (7.2–16.4), and 6.1 (2.7–13.6), respectively. As expected, sero-positivity for most IgA or IgG antibodies against the three HPyV antigens, was not associated with NPC risk (Figure 2).

In the two endemic areas, those who were tested IgA positive against BFRF1, LF2 and EBNA-1, or IgG positive against BMRF1, LF2 and BGLF2, had significantly increased risks of NPC. However, there were generally wide confidence intervals due to small numbers of cases included (Supplementary Figure S6, available as Supplementary data at *IJE* online). Similar, albeit less robust, risk estimates for above markers were also evident in non-endemic areas, in either sex or in people with or without a family history of cancer (Supplementary Figures S6–S8, available as Supplementary data at *IJE* online).

Both the two and four EBV-marker combinations showed strong NPC risk discriminatory ability in both models used, with overall C-statistics of 0.85 and 0.84, respectively. In general, the C-statistic values for both combinations were slightly lower in endemic than in non-endemic areas and in men than in women. However, the C-statistics tended to be higher among men from endemic areas than men from non-endemic areas, whereas the opposite was observed in women (Table 2;Supplementary Table S2, available as Supplementary data at *IJE* online). Based on the published algorithms, the adjusted HRs for NPC risk per unit increase of logit(*p*) were 1.49 (1.38–1.60) for the two EBV-marker combination and 1.76 (1.63–1.91) for the four EBV-marker combination (Supplementary Figure S9, available as Supplementary data at *IJE* online). Moreover, those who were IgG-positive against either or both of the LF2 and BGLF2 markers had significantly increased risk of NPC, with an adjusted HR of 27.7 (15.1–50.8) for double sero-positive individuals compared with double sero-negative individuals (Supplementary Table S3, available as Supplementary data at *IJE* online).

**Table 2.** C-statistic^a^ of two parsimonious EBV-marker combinations for predicting risk of NPC, overall and by time interval between blood sample collection and cancer diagnosis

EBV-marker combination	Endemic area	Non-endemic area	Overall
**Four EBV-marker combination** (BMRF1 IgA, LF2 IgA, BGLF2 IgG and LF2 IgG)			
Above cut-off for each marker			
Overall	0.799 (0.031)	0.844 (0.018)	0.843 (0.014)
Years since sample collection			
3–4	0.828 (0.034)	0.868 (0.019)	0.865 (0.016)
≥5	0.774 (0.054)	0.792 (0.032)	0.802 (0.026)
Based on previous algorithm^b^			
Overall	0.799 (0.032)	0.844 (0.017)	0.844 (0.014)
Years since sample collection			
3–4	0.817 (0.036)	0.866 (0.019)	0.865 (0.016)
≥5	0.778 (0.053)	0.784 (0.033)	0.801 (0.026)
**Two EBV-marker combination** (BGLF2 IgG and LF2 IgG)			
Above cut-off for each marker			
Overall	0.765 (0.033)	0.853 (0.017)	0.850 (0.014)
Years since sample collection			
3–4	0.795 (0.037)	0.877 (0.018)	0.869 (0.015)
≥5	0.779 (0.049)	0.805 (0.031)	0.813 (0.024)
Based on previous algorithm^c^			
Overall	0.778 (0.032)	0.851 (0.016)	0.847 (0.014)
Years since sample collection			
3–4	0.797 (0.036)	0.874 (0.018)	0.867 (0.015)
≥5	0.777 (0.048)	0.789 (0.032)	0.810 (0.025)

EBV, Epstein–Barr virus; NPC, nasopharyngeal cancer.

a Adjusted for age, sex, region and education, Standard errors are shown in brackets to facilitate comparisons between any pair of C-statistics in addition to allowing inference of the 95% confidence intervals (CIs).

b Algorithm for four EBV-marker combinations: logitp = –5.0015 + 3.4328 BMRF1IgA + 2.6795 LF2 IgA + 3.5153 BGLF2 IgG + 2.4180 LF2 IgG.

c Algorithm for two EBV-marker combination: logitp = –4.4302 + 4.4580 BGLF2 IgG + 3.9562 LF2 IgG.

In subgroup analyses defined by sex, area, endemic status, education, smoking or alcohol drinking, there was no evidence of significant heterogeneity in risk estimates, although those with a family history of cancer tended to have higher HRs than those without, for both parsimonious combinations. The increased NPC risk was also found higher among never-regular than ever-regular alcohol drinkers for the two EBV-marker combinations (Supplementary Figure S9, available as Supplementary data at *IJE* online). Additional adjustment for other risk factors did not materially alter the risk estimates, overall and in separate analyses by NPC endemic status (Supplementary Figure S10, available as Supplementary data at *IJE* online).

**Figure 3.**
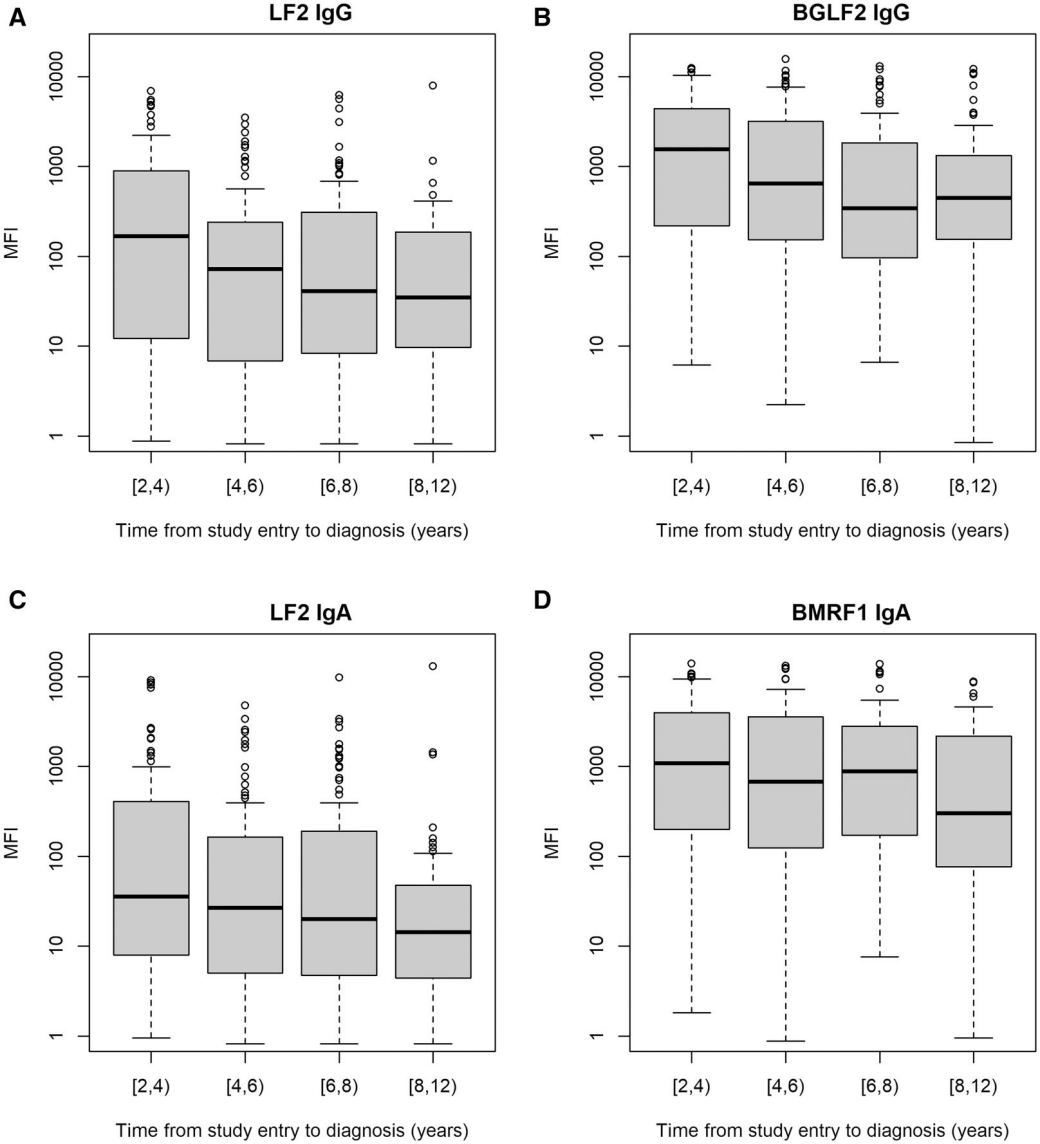
Boxplots of median fluorescence intensity values of four Epstein–Barr virus antibodies among nasopharyngeal carcinoma cases by the time interval between blood sample collection and nasopharyngeal cancer diagnosis. The solid horizontal lines are the median fluorescence intensity (MFI) for each time group. The box covers the first and third quartile of MFI. The whiskers extend to the most extreme data point which is no more than 1.5 times the interquartile range from the box

The MFI values of individual EBV antibodies appeared to decrease gradually with increased time interval between blood sample collection and cancer diagnosis among NPC cases (Figure 3; and Supplementary Figure S11, available as Supplementary data at *IJE* online). Consistent with this, in analyses by the time since sample collection, the HRs for NPC risk per unit increase of logit(*p*) associated with the four-marker and two-marker combinations also declined gradually, from 2.00 (1.77–2.25) and 1.66 (1.48–1.87) at the second year after blood draw to 1.61 (1.45–1.79) and 1.41 (1.29–1.55) after 8 years of follow-up, respectively (Table 3). Despite this, both EBV-marker combinations still had a strong discriminatory ability for NPC after 5 years since the blood draw, with C-statistics of around 0.8 for both (Table 2).

**Table 3.** Associations of two parsimonious EBV-marker combinations with risks of NPC, by the time interval between blood sample collection and cancer diagnosis

Years since study entry	No. of cases	HR (95% CI)^a^		
		Endemic area	Non-endemic area	Overall
**Four EBV-marker combination** (BMRF1 IgA, LF2 IgA, BGLF2 IgG and LF2 IgG)^b^				
3	43	1.86 (1.58–2.20)	2.03 (1.75–2.34)	2.00 (1.77–2.25)
4	29	2.01 (1.59–2.54)	1.69 (1.45–1.98)	1.80 (1.59–2.05)
5–6	81	1.80 (1.58–2.05)	1.76 (1.56–1.98)	1.79 (1.63–1.96)
7–8	81	1.70 (1.50–1.94)	1.62 (1.43–1.84)	1.66 (1.50–1.84)
>8	61	1.69 (1.46–1.96)	1.52 (1.31–1.76)	1.61 (1.45–1.79)
**Two EBV-marker combination** (BGLF2 IgG and LF2 IgG)^c^				
3	43	1.54 (1.29–1.84)	1.75 (1.51–2.04)	1.66 (1.48–1.87)
4	29	1.64 (1.30–2.07)	1.55 (1.34–1.79)	1.56 (1.38–1.76)
5–6	81	1.47 (1.26–1.72)	1.56 (1.40–1.74)	1.52 (1.38–1.66)
7–8	81	1.40 (1.20–1.63)	1.42 (1.28–1.58)	1.40 (1.28–1.53)
>8	61	1.52 (1.31–1.78)	1.34 (1.18–1.51)	1.41 (1.29–1.55)

EBV, Epstein–Barr virus; NPC, nasopharyngeal cancer; HRs, hazard ratios; CI, confidence interval.

a HRs for NPC risk per unit increase of logit(*p*), adjusted for age, sex, region and education.

b Algorithm for four EBV-marker combination: logitp = –5.0015 + 3.4328 BMRF1IgA + 2.6795 LF2 IgA + 3.5153 BGLF2 IgG + 2.4180 LF2 IgG.

c Algorithm for two EBV-marker combination: logitp = –4.4302 + 4.4580 BGLF2 IgG + 3.9562 LF2 IgG.

## Discussion

In this prospective evaluation of 16 EBV-markers and risk of NPC in Chinese adults from both NPC endemic and non-endemic regions, we demonstrated that, except for general infection marker VCAp18, IgA and IgG sero-positivity for the other 15 EBV antibodies were each significantly associated with higher risk of NPC. Moreover, two previously reported parsimonious combinations of EBV markers in clinical settings showed the strong discriminatory ability in reliably predicting NPC risk in the population setting. Importantly, despite declining slightly over time, the discriminatory ability was maintained after 5 years since blood collection in both NPC endemic and non-endemic areas.

Decades of epidemiological research have demonstrated consistently strong association between NPC risks and elevated antibody titres against antigens of VCA, EA and EBNA1, which only represent a very small fraction of the immune response against the nearly 100 proteins expressed by EBV.^14^^,^^19^^,^^29^ Based on a small prospective study of >2000 participants with 34 cases of NPC, measurements of circulating cell-free EBV DNA have also been suggested as an alternative approach for detecting early asymptomatic NPC cases.^30^ However, with no substantial improvement on the screening performance, measurement of circulating EBV DNA is more expensive, with complex laboratory infrastructure/skills required compared with serological assays.^31^ Moreover, a prospective study in Singapore directly compared both EBV serology and serum cell-free DNA as screening tools for NPC among the same 524 participants and reported better performance of serology detecting overall and asymptomatic early-stage NPC cases compared with DNA.^32^

In a case-control study (175 cases and 175 controls) in Taiwan,^23^ the Luminex bead-based multiplex serology, as used in the present study, has fully adapted/validated multiple NPC-specific EBV-markers. These have been identified by a peptide-based whole-proteome microarray technique with a significant improvement in accuracy beyond the current NPC screening programme used EBV biomarkers.^22^ At 95% specificity, the sensitivity for all cases and for stage I/IIa NPC cases was 98.8% and 100%, respectively. Moreover, two further refined combinations of four and two EBV-antibodies identified were capable of confirming clinically diagnosed NPC with ∼99% accuracy, and detecting early-stage NPC cases with 98.5% sensitivity at 95% specificity.^23^ In addition to the generally consistent findings at the individual antibody level, the present study has further demonstrated that the two IgG EBV-marker combination performed as well as the four EBV-marker combination that involved both IgG and IgA markers, even more than 5 years before cancer diagnosis. Without testing IgA simultaneously, the two IgG EBV-marker combination can reduce the number of reactions, greatly facilitating implementation in practice. Therefore, the IgG-based antibody test against LF2 and BGLF2 could be considered as an optimal mass-screening test for NPC in future. In our study, at either 97% or 95% specificity, the sensitivity was ∼50% in both endemic and non-endemic areas but decreased with increasing time since the sample collection; the positive predictive value (PPV) was high, particularly in endemic areas (>90%) (Supplementary Table S4, available as Supplementary data at *IJE* online). However, due to the case-cohort study design with exclusion of the first 2 years of follow-up, the sensitivity could well be underestimated, whereas PPV could be overestimated. Further validation study in an independent cohort from different populations and settings, including a direct comparison with current serology screening assay in the same populations, will further assess and strengthen the robustness of the present findings.

The specific roles of the proteins LF2 and BGLF2 (both are EBV early proteins), and particularly their contribution to NPC development, are less well understood. BGLF2 is a tegument protein that is important for the morphogenesis of virions, and can induce the expression of BZLF1 during latency and protect cells from α-interferon, both mechanisms that can enhance EBV virion production.^33^ LF2 has attracted attention because of its ability to bind to Rta (BRLF1)^34^ and may inhibit EBV replication by altering the Rta location within the cell.^35^^,^^36^

The present study has a number of strengths, including the prospective nature of the sample collection, large numbers of NPC cases from both endemic and non-endemic regions in China, comprehensive assessment of both IgA and IgG antibodies against 16 EBV antigens, and measurement of HPyVs antibodies for control purposes. Moreover, as part of the study design, we excluded the first 2 years since sample collection to avoid including prevalent yet undiagnosed cases and minimize potential reverse causation bias. However, the study also has limitations. First, although it is by far the largest prospective study of EBV markers and NPC risk, the study currently still lacks adequate power to reliably investigate the relevance of different antigen combinations and the associations in certain population subgroups. Second, although various NPC risk factors were adjusted in the model, no information was collected on consumption of Chinese-style salt-preserved fish which has been recognized as a risk factor for NPC in southern China.^37^ However, a recently published review reported a diminishing role of salt-preserved fish in NPC development in China, due to significantly reduced intake in recent decades as a result of improvements in living standards and dietary patterns.^3^ Third, our study was not nationally representative. However, the standardized NPC incidence rate from 10 diverse areas (including two from NPC endemic regions) in CKB was only slightly higher than that in the general Chinese population (8.6 vs 7.3 per 100 000^38^). Finally, we were not able to assess the roles of genetic, molecular risk factors, coinfection or host antibody-mediated immune response to EBV infection in NPC aetiology, so residual confounding may still exist.

## Conclusion

In summary, this prospective study demonstrated strong associations of multiple EBV-markers with risk of NPC in Chinese adults. Moreover, the occurrence of NPC can be reliably predicted using LF2/BGLF2 IgG combination for more than 5 years. Our study demonstrated that regular screening of high-risk NPC individuals using a simple serology test may offer an optimal and cost-effective approach for NPC early detection in clinical and general community settings, in both endemic and non-endemic areas.

## Ethics approval

The China Kadoorie Biobank (CKB) complies with all the required ethical standards for medical research on human subjects. Ethical approvals were granted and have been maintained by the relevant institutional ethical research committees in the UK and China. Informed consent was obtained from all participants included in the study.

## Supplementary Material

dyae098_Supplementary_Data

## Data Availability

Anonymized baseline, resurvey, sample assay and cause-specific mortality and morbidity data are available for access through a formal application in the CKB website [www.ckbiobank.org]. The application will then be reviewed by a Data Access Committee. Further details about access policy and procedures can be found online at [www.ckbiobank.org].
