# Supplementary material for: Prospective evaluation of the relevance of Epstein–Barr virus antibodies for early detection of nasopharyngeal carcinoma in Chinese adults
Source: Int J Epidemiol. 2024 Jul 15;53(4):dyae098. doi: 10.1093/ije/dyae098 (PMC11249388; doi:10.1093/ije/dyae098)
Supplement: dyae098_Supplementary_Data [file dyae098_supplementary_data.docx]

**Prospective evaluation of the relevance of Epstein-Barr virus antibodies for early detection of nasopharyngeal carcinoma in Chinese adults**

**Supplementary materials**

**Contents**

[**Members of the China Kadoorie Biobank collaborative group:** 2](#_Toc144885588)

[**Methods:** 3](#_Toc144885589)

[**Table S1. Cut-off values of IgA and IgG antibodies for each EBV marker** 5](#_Toc144885590)

[**Table S2. C-statistics of the two parsimonious EBV-marker combinations for predicting risk of NPC by sex, overall and by time since blood sample collection** 6](#_Toc144885591)

[**Table S3. Adjusted HRs for NPC by different combinations of IgG antibodies against LF2 and BGLF2 markers** 7](#_Toc144885592)

# **Table S4. Performance of EBV-marker combinations for NPC screening, overall and by NPC endemic status or by year since sample collection……………………………………….** 8

[**Figure S1. Locations of the 10 survey areas and number recruited.** 9](#_Toc144885593)

[**Figure S2. Flow diagram of study design and participant selection** 10](#_Toc144885594)

[**Figure S3. Age-standardized cumulative incidence rates of NPC in CKB by (A) sex, (B) area, (C) sex and endemic/non-endemic area** 11](#_Toc144885595)

[**Figure S4. Distribution of IgA and IgG antigens’ MFI value in the subcohort** 12](#_Toc144885596)

[**Figure S5. Kendall’s** $\tau$ **correlation coefficients of EBV markers (MFI values) among subcohort participants** 13](#_Toc144885597)

[**Figure S6. Adjusted HRs for NPC associated with sero-positivity of specific EBV antibodies in endemic and non-endemic areas** 14](#_Toc144885599)

[**Figure S7. Adjusted HRs for NPC associated with sero-positivity of specific EBV antibodies in men and women** 15](#_Toc144885601)

[**Figure S8: Adjusted HRs for NPC associated with sero-positivity of specific EBV antibodies by family history of cancer** 16](#_Toc144885603)

[**Figure S9. Adjusted HRs for NPC associated with two parsimonious EBV-marker combinations, in selected subgroups** 17](#_Toc144885604)

[**Figure S10. Adjusted HRs for NPC associated with two parsimonious EBV-marker combinations, in analyses with further adjustment for other risk factors** 18](#_Toc144885605)

[**Figure S11. Distribution of MFI values of each EBV antigen among cases by the time interval between blood sample collection and cancer diagnosis** 19](#_Toc144885606)

# **Members of the China Kadoorie Biobank collaborative group:**

**International Steering Committee:** Junshi Chen, Zhengming Chen (PI), Robert Clarke, Rory Collins, Yu Guo, Liming Li (PI), Chen Wang, Jun Lv, Richard Peto, Robin Walters.

**International Co-ordinating Centre, Oxford:** Daniel Avery, Derrick Bennett, Ruth Boxall, Ka Hung Chan, Yiping Chen, Zhengming Chen, Johnathan Clarke; Robert Clarke, Huaidong Du, Ahmed Edris Mohamed, Hannah Fry, Simon Gilbert, Mike Hill, Pek Kei Im, Andri Iona, Maria Kakkoura, Christiana Kartsonaki, Kuang Lin, Mohsen Mazidi, Iona Millwood, Sam Morris, Qunhua Nie, Alfred Pozarickij, Paul Ryder, Saredo Said, Dan Schmidt, Paul Sherliker, Rajani Sohoni, Becky Stevens, Iain Turnbull, Robin Walters, Lin Wang, Neil Wright, Ling Yang, Xiaoming Yang, Pang Yao.

**National Co-ordinating Centre, Beijing:** Yu Guo, Xiao Han, Can Hou, Qingmei Xia, Chao Liu, Jun Lv, Pei Pei, Canqing Yu.

**10 Regional Co-ordinating Centres:**

**Guangxi** Provincial CDC: Naying Chen, Duo Liu, Zhenzhu Tang. Liuzhou CDC: Ningyu Chen, Qilian Jiang, Jian Lan, Mingqiang Li, Yun Liu, Fanwen Meng, Jinhuai Meng, Rong Pan, Yulu Qin, Ping Wang, Sisi Wang, Liuping Wei, Liyuan Zhou. **Gansu** Provincial CDC: Caixia Dong, Pengfei Ge, Xiaolan Ren. Maiji CDC: Zhongxiao Li, Enke Mao, Tao Wang, Hui Zhang, Xi Zhang. **Hainan** Provincial CDC: Jinyan Chen, Ximin Hu, Xiaohuan Wang. Meilan CDC: Zhendong Guo, Huimei Li, Yilei Li, Min Weng, Shukuan Wu. **Heilongjiang** Provincial CDC: Shichun Yan, Mingyuan Zou, Xue Zhou. Nangang CDC: Ziyan Guo, Quan Kang, Yanjie Li, Bo Yu, Qinai Xu. **Henan** Provincial CDC: Liang Chang, Lei Fan, Shixian Feng, Ding Zhang, Gang Zhou. Huixian CDC: Yulian Gao, Tianyou He, Pan He, Chen Hu, Huarong Sun, Xukui Zhang. **Hunan** Provincial CDC: Biyun Chen, Zhongxi Fu, Yuelong Huang, Huilin Liu, Qiaohua Xu, Li Yin. Liuyang CDC: Huajun Long, Xin Xu, Hao Zhang, Libo Zhang. **Jiangsu** Provincial CDC: Jian Su, Ran Tao, Ming Wu, Jie Yang, Jinyi Zhou, Yonglin Zhou. Suzhou CDC: Yihe Hu, Yujie Hua, Jianrong Jin Fang Liu, Jingchao Liu, Yan Lu, Liangcai Ma, Aiyu Tang, Jun Zhang. **Qingdao** Qingdao CDC: Liang Cheng, Ranran Du, Ruqin Gao, Feifei Li, Shanpeng Li, Yongmei Liu, Feng Ning, Zengchang Pang, Xiaohui Sun, Xiaocao Tian, Shaojie Wang, Yaoming Zhai, Hua Zhang, Licang CDC: Wei Hou, Silu Lv, Junzheng Wang. **Sichuan** Provincial CDC: Xiaofang Chen, Xianping Wu, Ningmei Zhang, Weiwei Zhou. Pengzhou CDC: Xiaofang Chen, Jianguo Li, Jiaqiu Liu, Guojin Luo, Qiang Sun, Xunfu Zhong. **Zhejiang** Provincial CDC: Weiwei Gong, Ruying Hu, Hao Wang,Meng Wan, Min Yu. Tongxiang CDC: Lingli Chen, Qijun Gu, Dongxia Pan，Chunmei Wang, Kaixu Xie, Xiaoyi Zhang.

# **Methods:**

**Sample preparation and antibodies selection for EBV serology assay:**

The stored baseline plasma samples from cases and subcohort participants were retrieved, thawed, subaliquoted and plated at the Wolfson laboratory, University of Oxford, and were then shipped on dry ice to the laboratory at the German Cancer Research Center (DKFZ).

Plasma samples were pre-incubated at 1:50 dilution for IgA testing (final dilution 1:100) and at 1:5000 dilution for IgG testing (final dilution 1:10 000) in a pre-incubation buffer based on phosphate-buffered saline (PBS) with 2 mg/mL casein and additionally containing 2g/L of lysate proteins of *Escherichia coli* overexpressing GST-tag, 5g/L polyvinyl alcohol and 8g/L polyvinyl-pyrrolidone.(34) Bound antibodies were detected with goat anti-Human IgG-Biotin (1:1000, Jackson ImmunoResearch, West Grove, Pennsylvania) and goat anti-Human IgA-Biotin (1:1000, #109-065-011, Jackson Immuno Research), respectively, and subsequently stained with streptavidin-R-phycoerythrin (1:750, MOSS Inc., Elk Grove Village, Illinois).

The 16 EBV antigens assayed included EBV general infection markers (Zebra, EBNA1-trunc, BMRF1 (EA-D), VCAp18 and EBNA1-peptide) ^1^, EBV antigens previously identified specific for NPC (LF2, BZLF1 (Zebra-trunc, including 43 amino acids), BGLF2, BRLF1, BFRF1, BORF1, BXLF1) ^2^ and EBV antigens derived from an array-based approach for identifying EBV-positive classical Hodgkin lymphoma (VCAp40, BaRF1, BHRF1, BBRF1) ^3^. Compare with the 13-marker set included in the previous serology been validated in the case-control study ^2^, one antigen (BPLF1 IgA) was dropped in the present study due to the technical measurement problem, however, the performance calculated based on the 4-marker performance was equally as shown previously in the validation study ^2^. Furthermore, three antigens representing the major capsid protein VP1 of the three human polyomaviruses (HPyV), i.e. JC, BK and HPyV6 were also included as quality control markers (i.e. with no association expected with NPC).

**Reference:**

1. Brenner N, Mentzer AJ, Butt J, et al. Validation of Multiplex Serology detecting human herpesviruses 1-5. *PLoS One*. 2018;13(12):e0209379.
2. Simon J, Liu Z, Brenner N, et al. Validation of an Epstein-Barr Virus Antibody Risk Stratification Signature for Nasopharyngeal Carcinoma by Use of Multiplex Serology. *J Clin Microbiol*. 2020;58(5):e00077-20.
3. Liu Z, Jarrett RF, Hjalgrim H, et al. Evaluation of the antibody response to the EBV proteome in EBV-associated classical Hodgkin lymphoma. *Int J Cancer*. 2020;147(3):608-618.

# **Table S1. Cut-off values of IgA and IgG antibodies for each EBV marker**

| **EBV marker** | **IgA** | **IgG** |
| --- | --- | --- |
| EBNA truncated | 1809 | 12664 |
| EBNA-1 | 1421 | 6752 |
| BRLF1 | 1088 | 212 |
| BZLF1 | 305 | 39 |
| Zebra | 1278 | 1631 |
| BHRF1 | 2000 | 3300 |
| BMRF1 | 3484 | 1837 |
| BXLF1 | 1155 | 645 |
| BaRF1 | 2000 | 1000 |
| BFRF1 | 296 | 347 |
| BGLF2 | 885 | 626 |
| VCA p18 | 8414 | 7478 |
| VCAp40 | 1800 | 1000 |
| BORF1 | 1559 | 202 |
| BBRF1 | 160 | 120 |
| LF2 | 92 | 36 |
| BK VP1 | 400 | 250 |
| JC VP1 | 400 | 250 |
| HpyV6 VP1 | 400 | 250 |

Abbreviations: EBV - Epstein-Barr virus

#

# **Table S2. C-statistics of the two parsimonious EBV-marker combinations for predicting risk of NPC by sex, overall and by time since blood sample collection**

| **EBV-markers combination** | **Males** | | | **Females** | | |
| --- | --- | --- | --- | --- | --- | --- |
|  | **Endemic area** | **Non-endemic area** | **Overall** | **Endemic area** | **Non-endemic area** | **Overall** |
| **a) Four EBV-marker** (BMRF1 IgA, LF2 IgA, BGLF2 IgG and LF2 IgG) | | | |  |  |  |
| **Above cut-off for each marker** |  |  | |  |  |  |
| Overall | 0.842 (0.040) | 0.821 (0.025) | 0.829 (0.021) | 0.759 (0.052) | 0.855 (0.026) | 0.854 (0.020) |
| Years since sample collection |  |  |  |  |  |  |
| 3-4 | 0.856 (0.042) | 0.859 (0.026) | 0.859 (0.022) | 0.811 (0.055) | 0.867 (0.030) | 0.861 (0.024) |
| ≥ 5 | 0.848 (0.053) | 0.785 (0.041) | 0.794 (0.035) | 0.835 (0.057) | 0.864 (0.041) | 0.870 (0.029) |
| **Based on previous algorithm** ^#^ |  |  | |  |  |  |
| Overall | 0.829 (0.041) | 0.818 (0.025) | 0.832 (0.020) | 0.778 (0.048) | 0.848 (0.027) | 0.849 (0.021) |
| Years since sample collection |  |  |  |  |  |  |
| 3-4 | 0.822 (0.048) | 0.853 (0.026) | 0.848 (0.024) | 0.781 (0.063) | 0.854 (0.032) | 0.840 (0.022) |
| ≥ 5 | 0.846 (0.053) | 0.765 (0.043) | 0.868 (0.024) | 0.845 (0.053) | 0.844 (0.043) | 0.851 (0.021) |
| **b)Two EBV-marker** (BGLF2 IgG and LF2 IgG) | | | |  |  |  |
| **Above cut-off for each marker** |  |  |  |  |  |  |
| Overall | 0.842 (0.040) | 0.826 (0.025) | 0.828 (0.021) | 0.759 (0.052) | 0.866 (0.024) | 0.860 (0.019) |
| Years since sample collection |  |  |  |  |  |  |
| 3-4 | 0.776 (0.056) | 0.858 (0.026) | 0.849 (0.022) | 0.754 (0.062) | 0.868 (0.029) | 0.863 (0.023) |
| ≥ 5 | 0.838 (0.053) | 0.772 (0.044) | 0.797 (0.035) | 0.840 (0.052) | 0.856 (0.041) | 0.869 (0.028) |
| **Based on previous algorithm ^$^** |  |  |  |  |  |  |
| Overall | 0.772 (0.049) | 0.822 (0.025) | 0.828 (0.021) | 0.791 (0.044) | 0.865 (0.024) | 0.860 (0.019) |
| Years since sample collection |  |  |  |  |  |  |
| 3-4 | 0.768 (0.055) | 0.852 (0.027) | 0.855 (0.021) | 0.761 (0.062) | 0.868 (0.029) | 0.841 (0.021) |
| ≥ 5 | 0.831 (0.054) | 0.772 (0.041) | 0.866 (0.024) | 0.841 (0.052) | 0.847 (0.040) | 0.854 (0.020) |

* Adjusted for age, sex, region, and education

^#^ Algorithm for 4 EBV-marker model: logitp = –5.0015 + 3.4328 EA-D IgA + 2.6795 LF2 IgA + 3.5153 BGLF2 IgG + 2.4180 LF2 IgG;

^$^ Algorithm for 2 EBV-marker model: logitp = -4.4302 + 4.4580 BGLF2 IgG + 3.9562 LF2 IgG

Abbreviations: EBV - Epstein-Barr virus; NPC – Nasopharyngeal cancer

# **Table S3. Adjusted HRs for NPC by different combinations of IgG antibodies against LF2 and BGLF2 markers**

| **LF2 IgG** | **BGLF2 IgG** | **N (%) in Subcohort** | **HR (95%CI)*** |
| --- | --- | --- | --- |
| **-** | **-** | 632 (85.5) | 1.00 (Reference) |
| **-** | **+** | 44 (6.0) | 5.22 (3.06, 8.91) |
| **+** | **-** | 44 (6.0) | 3.18 (1.71, 5.93) |
| **+** | **+** | 19 (2.6) | 27.71 (15.11, 50.82) |

* Model was adjusted for age (continuous), sex, area (10 areas), and education (≥6 years).

Abbreviations: EBV - Epstein-Barr virus; NPC – Nasopharyngeal cancer; HR – Hazard ratio; CI – Confidence interval

# **Table S4. Performance of EBV-marker combinations for NPC screening, overall and by NPC endemic status or by year since sample collection**

|  | **Specificity 97%** | | | |  | **Specificity 95%** | | | | |  |
| --- | --- | --- | --- | --- | --- | --- | --- | --- | --- | --- | --- |
|  | **Sensitivity** | | **PPV** | |  | **Sensitivity** | | | **PPV** | |  |
| **Overall** | |  | |  | | |  |  | |  | |
| Four EBV-marker * | 48.6 | | 85.5 | |  | 54.8 | | | 70.5 | |  |
| Two EBV-marker # | 47.3 | | 68.7 | |  | 47.3 | | | 68.7 | |  |
| **NPC endemic status** | |  | |  | | |  |  | |  | |
| **Endemic area** | |  | |  | | |  |  | |  | |
| Four EBV-marker * | 52.8 | | 91.8 | |  | 57.5 | | | 91.0 | |  |
| Two EBV-marker # | 47.2 | | 90.9 | |  | 54.7 | | | 81.7 | |  |
| **Non-endemic area** | |  | |  | | |  |  | |  | |
| Four EBV-marker * | 50.0 | | 63.7 | |  | 50.0 | | | 63.7 | |  |
| Two EBV-marker # | 43.0 | | 61.5 | |  | 55.1 | | | 61.5 | |  |
| **Years since sample collection** | |  | |  | | |  |  | |  | |
| **3-4** | |  | |  | | |  |  | |  | |
| Four EBV-marker * | 55.1 | | 81.8 | |  | 61.2 | | | 64.1 | |  |
| Two EBV-marker # | 52.6 | | 62.0 | |  | 52.6 | | | 62.0 | |  |
| **≥5** | |  | |  | | |  |  | |  | |
| Four EBV-marker * | 35.4 | | 59.6 | |  | 41.7 | | | 38.1 | |  |
| Two EBV-marker # | 36.5 | | 36.5 | |  | 36.5 | | | 36.5 | |  |

*: Four EBV-marker: include BMRF1 IgA, LF2 IgA, BGLF2 IgG and LF2 IgG;

#: Two-EBV-marker: include BGLF2 IgG and LF2 IgG

Abbreviations: EBV - Epstein-Barr virus; NPC – Nasopharyngeal cancer; PPV: Positive predictive value;

Please note: Given the study design, the sensitivity would be expected to be lower than would have been if the first two years had been included, when individuals with existing yet undiagnosed NPC would have been picked up. On the contrary, the PPV may be higher than it would have been in the general population, thus the results need to be interpreted with caution.

#

# **Figure S1. Locations of the 10 survey areas and number recruited.**

Open circles indicate rural areas and solid circles indicate urban areas. Number recruited at baseline in each area is shown in brackets.


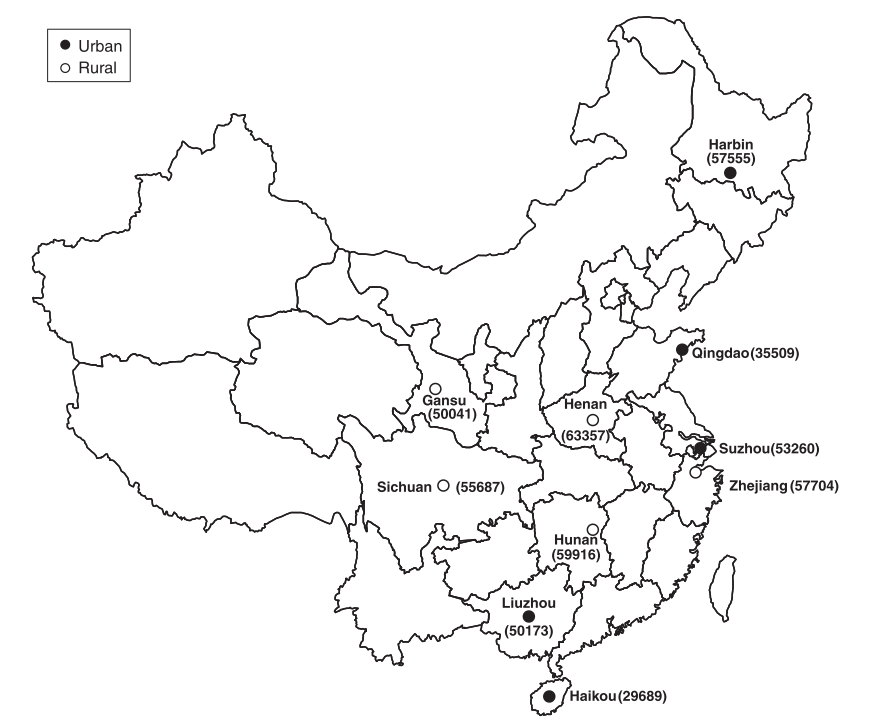


# **Figure S2. Flow diagram of study design and participant selection**

*Selection for the subcohort was done using simple random sampling; †Individuals may be included in more than one study arm;

Abbreviations: CKB: China Kadoorie Biobank; NPC: nasopharyngeal cancer.

All CKB participants

n=512,715, total NPC=370

Eligible CKB participants

n=452,957

Had cancer history at baseline

n=2,411

Cancer event or died in first 2 years follow-up

n=6,150

Genotyped participants

n=65,684

Not genotyped, or selection for genotyping not random

n=387,273

**Study arm 2^†^**

Subcohort

n=746

Not selected* n=65,184

Sample been reformatted or with other known problems

n=51,197

**Study arm 1^†^**

NPC cases

n=295

Subcohort

n=2000

# **Figure S3. Age-standardized cumulative incidence rates of NPC in CKB by (A) sex, (B) area, (C) sex and endemic/non-endemic area**

*
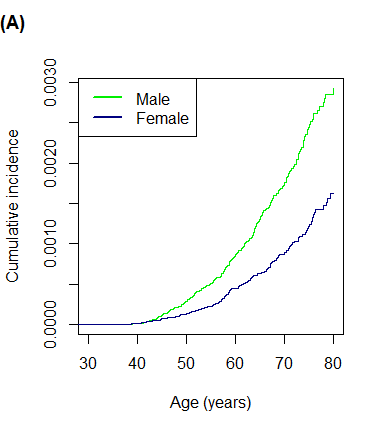
* **
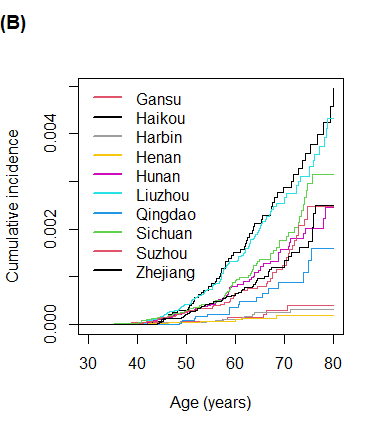

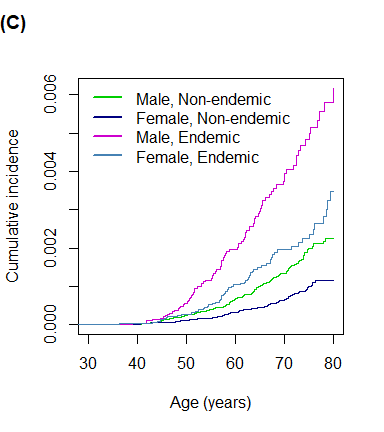
**

Abbreviations: CKB - China Kadoorie Biobank; NPC – Nasopharyngeal cancer

# **Figure S4. Distribution of IgA and IgG antigens’ MFI value in the subcohort**

The pink line indicates the cut-off value of the median fluorescence intensity (MFI) assigned for each antigen


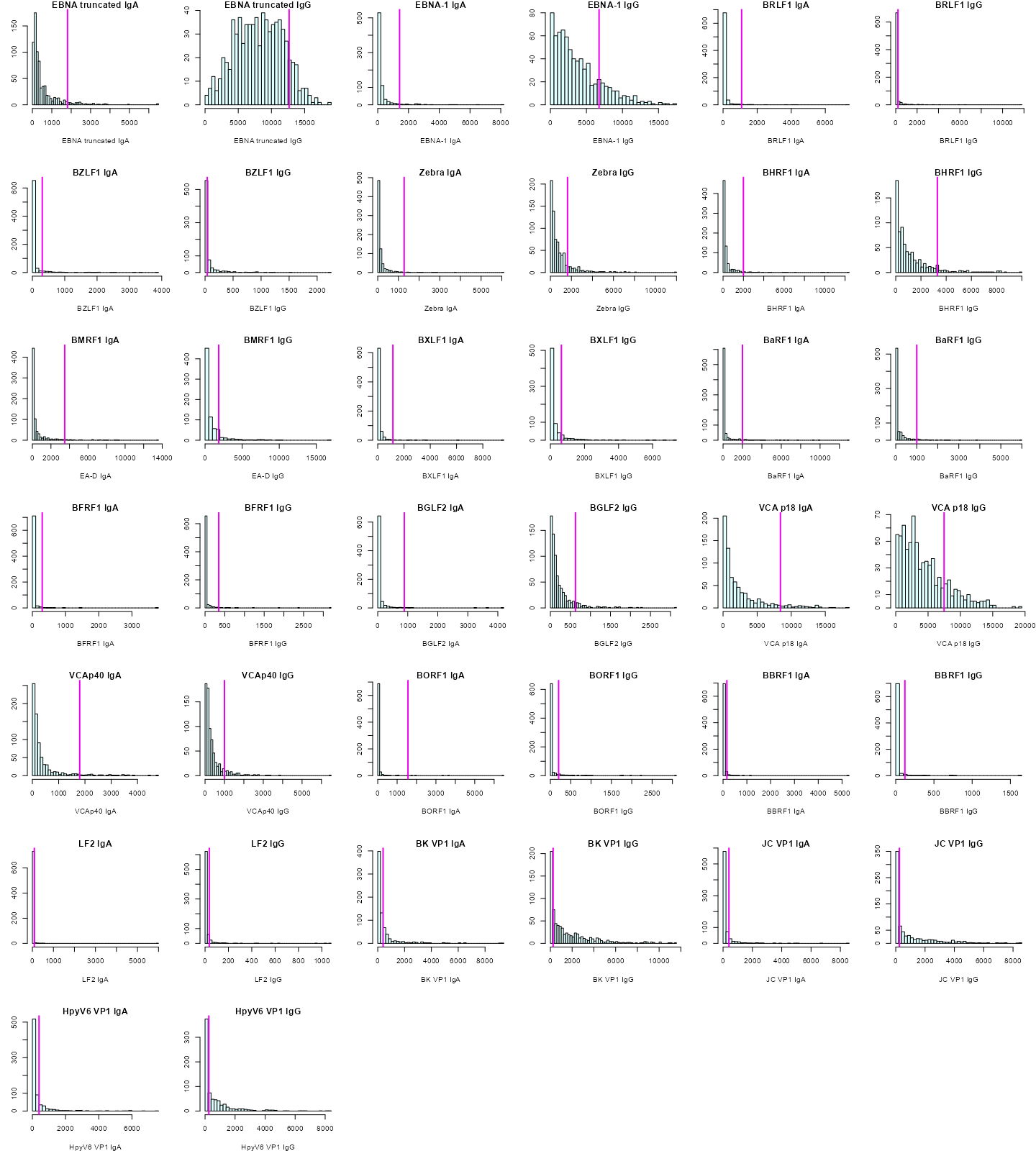


# **Figure S5. Kendall’s** $\boldsymbol{\tau}$ **correlation coefficients of EBV markers (MFI values) among subcohort participants**

#
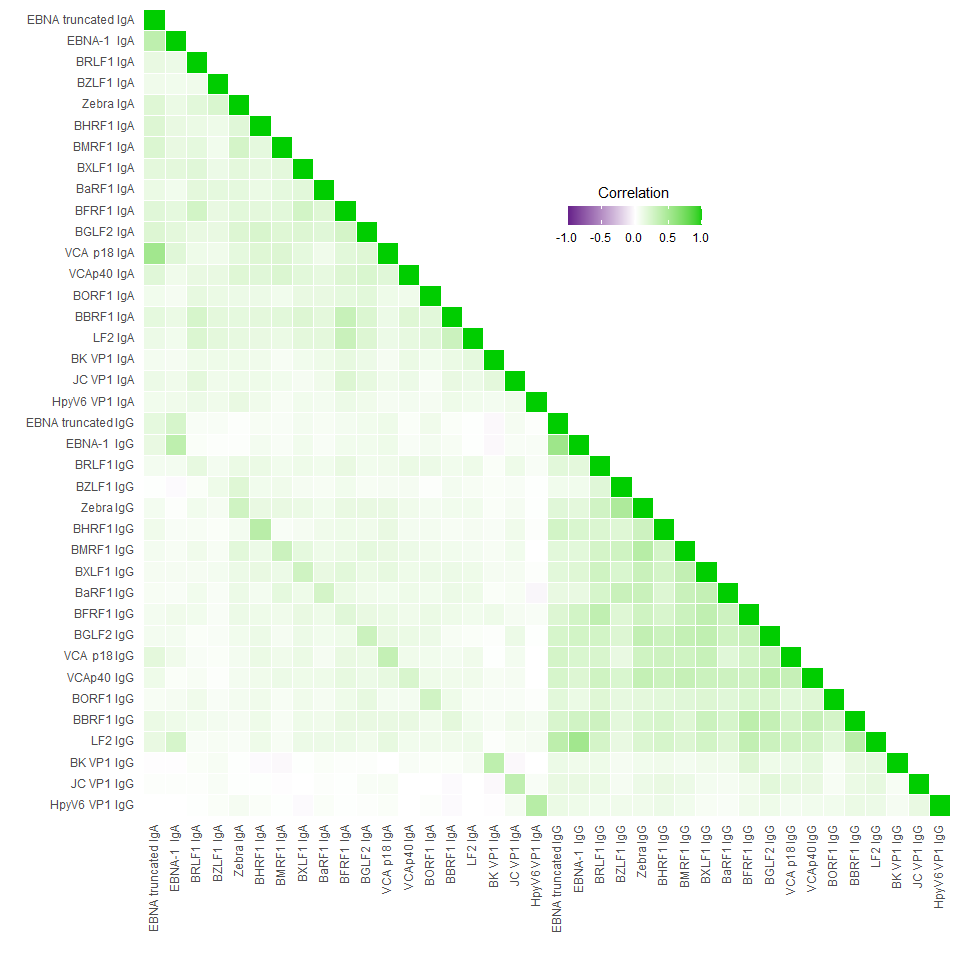


Abbreviations: EBV - Epstein-Barr virus; MFI - median fluorescence intensity

# **Figure S6. Adjusted HRs for NPC associated with sero-positivity of specific EBV antibodies in endemic and non-endemic areas**

(A) for 8 non-endemic areas and (B) for 2 endemic areas. Models were adjusted for age, sex, area, and education. The boxes indicate HRs for different EBV markers (solid for IgA and open for IgG antibodies), with the area of each box inversely proportional to the variance of the logHR. The horizontal lines represent 95% CIs. Note: No risk estimate was obtained for BGLF2 IgA and BRLF1 IgA in endemic areas due to none sample was sero-positive among the subcohort group. Abbreviations: HRs – hazard ratios; NPC – nasopharyngeal cancer; EBV - Epstein-Barr virus


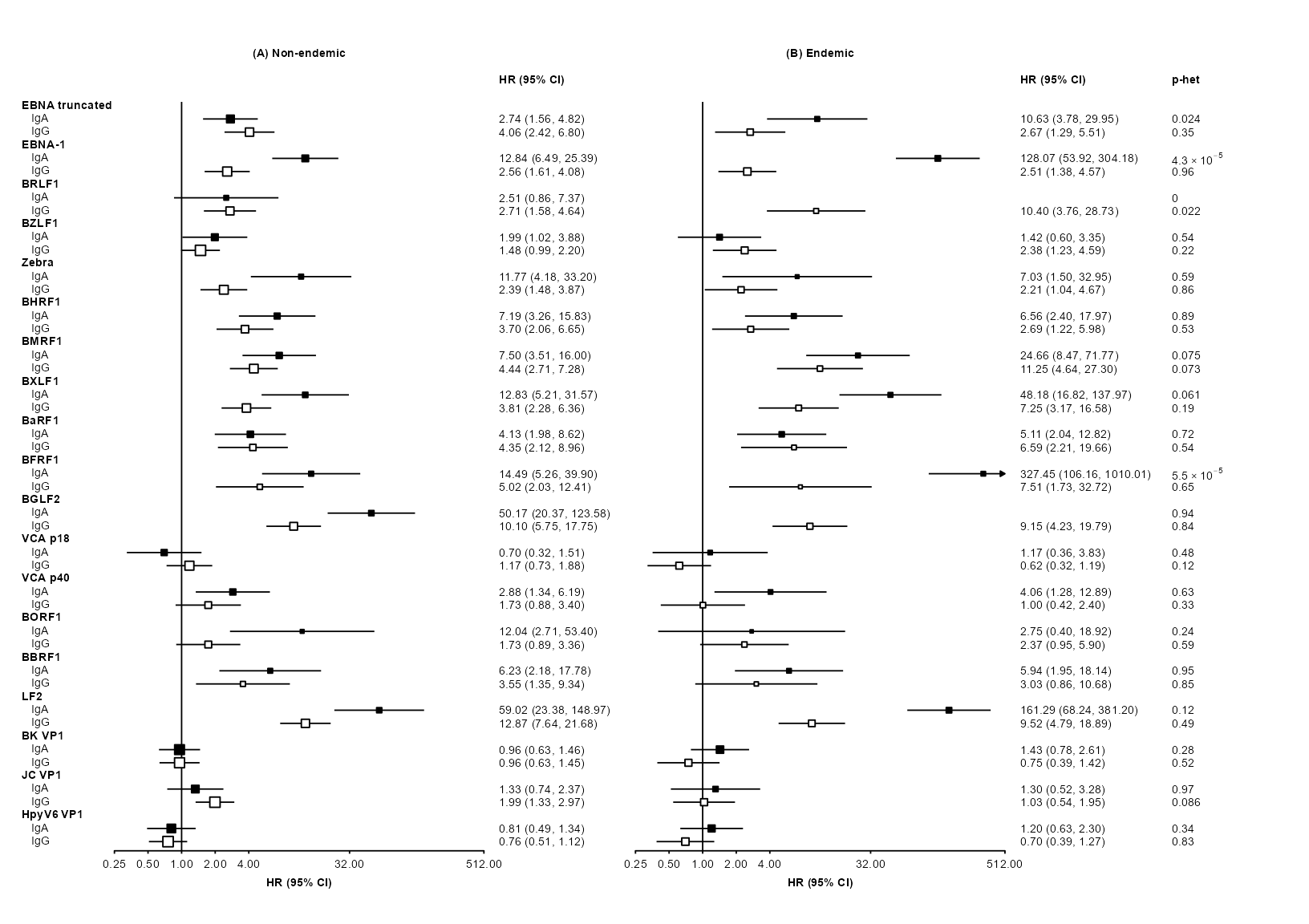


# **Figure S7. Adjusted HRs for NPC associated with sero-positivity of specific EBV antibodies in men and women**

1. for male participants and (B) for female participants. Convention as in Figure S6.

Abbreviations: HRs – hazard ratios; NPC – nasopharyngeal cancer; EBV - Epstein-Barr virus

**
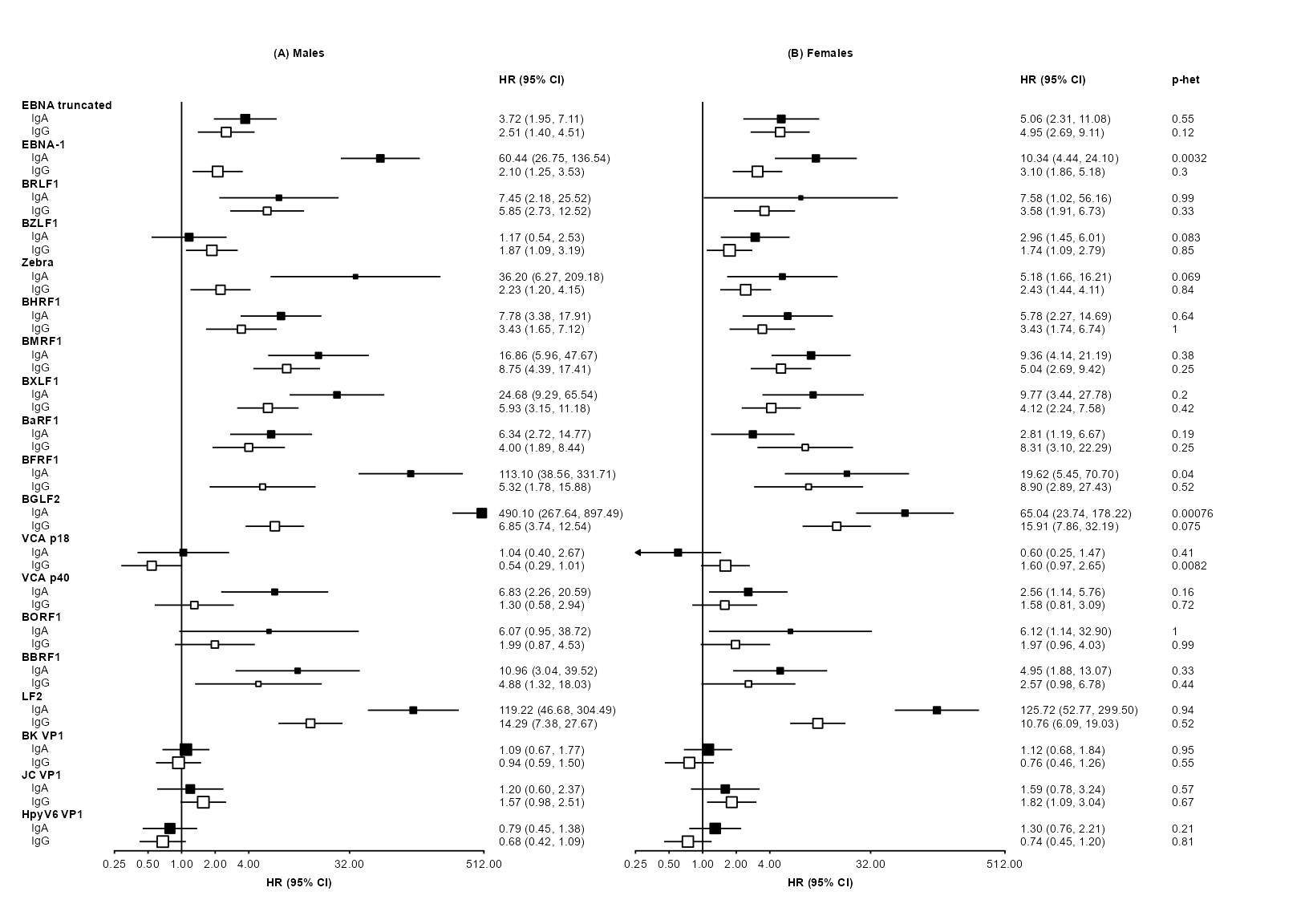
**

# **Figure S8: Adjusted HRs for NPC associated with sero-positivity of specific EBV antibodies by family history of cancer**

(A) for participants without family history of cancer and (B) for participants with family history of cancer. Convention as in Figure S6. Abbreviations: HRs – hazard ratios; NPC – nasopharyngeal cancer; EBV - Epstein-Barr virus

**
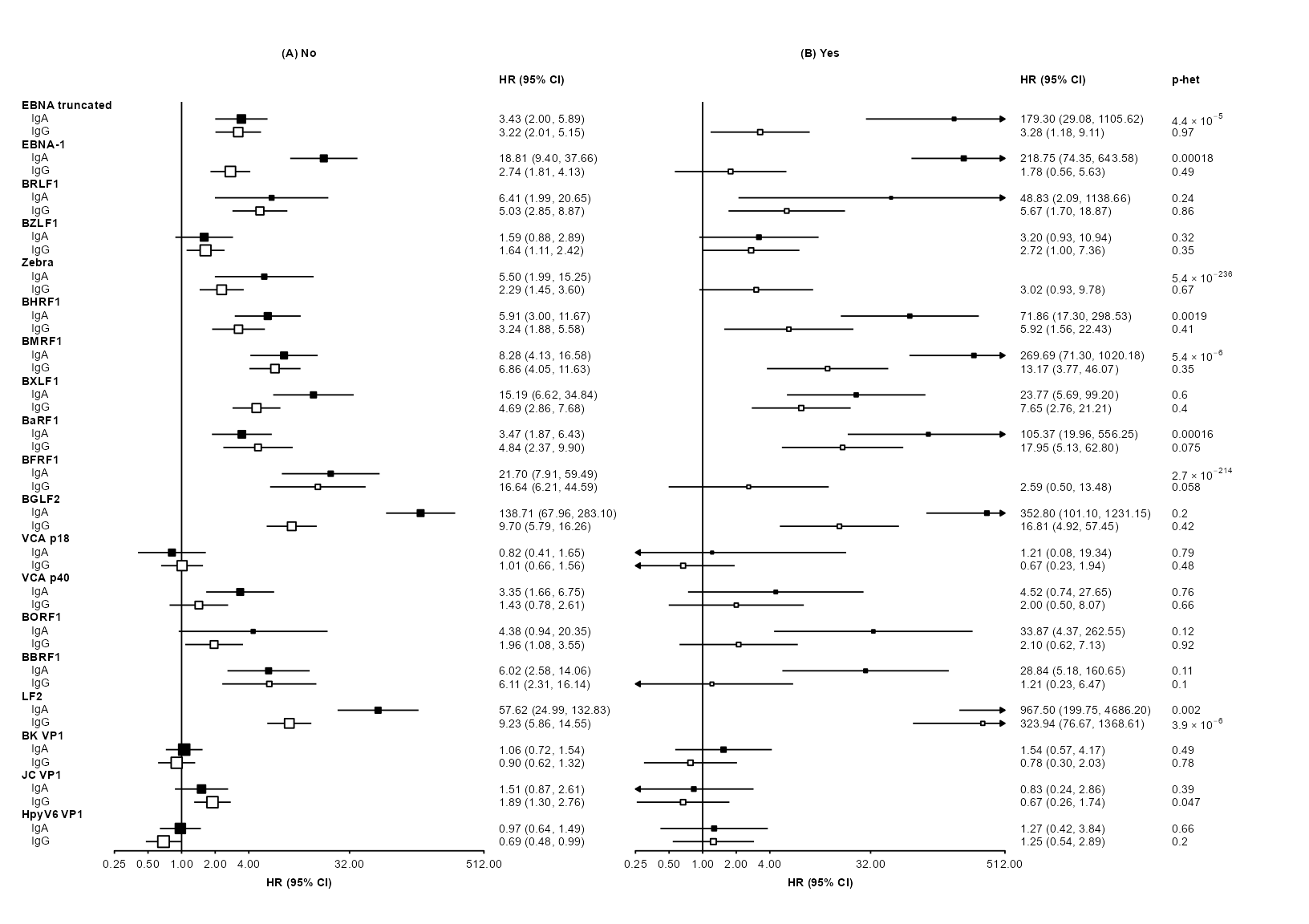
**

# **Figure S9. Adjusted HRs for NPC associated with two parsimonious EBV-marker combinations, in selected subgroups**

Models were adjusted for age, sex, area, and education. The boxes indicate HRs for each parsimonious EBV-marker combination in each subgroup population, with the area of each box inversely proportional to the variance of the logHR. The horizontal lines represent 95% CIs. The diamond indicates the overall HR for each marker combination. Abbreviations: HRs – hazard ratios; NPC – nasopharyngeal cancer; EBV - Epstein-Barr virus


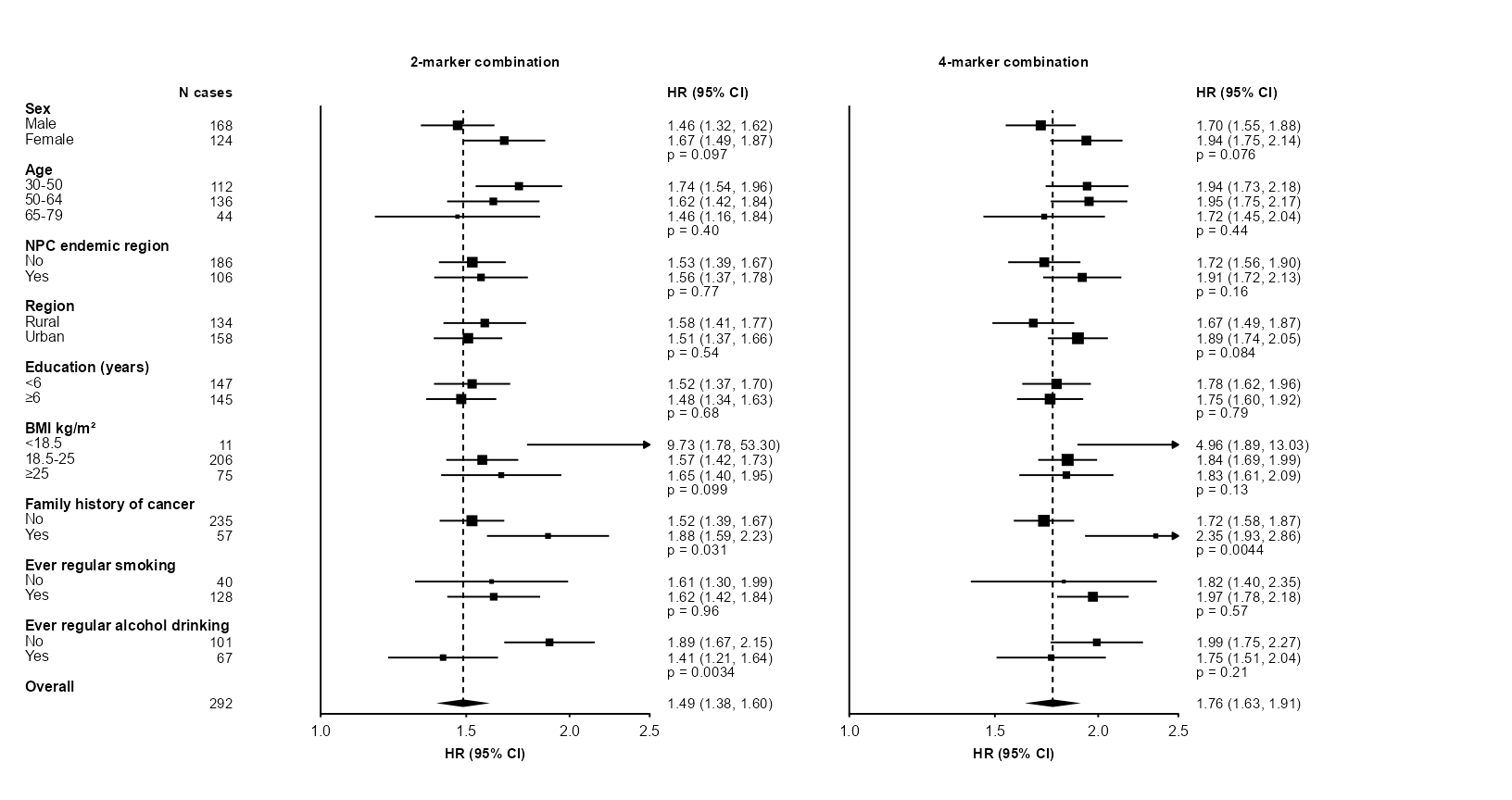


# **Figure S10. Adjusted HRs for NPC associated with two parsimonious EBV-marker combinations, in analyses with further adjustment for other risk factors**

(A)for overall areas; (B) for 8 non-endemic areas and (C) for 2 endemic-areas. Each closed square represents a change in adjusted HRs for NPC risk per unit increase of logit(*p*); Abbreviations: HRs – hazard ratios; NPC – nasopharyngeal cancer; EBV - Epstein-Barr virus

**(A) Overall areas (n=10 areas)**

**
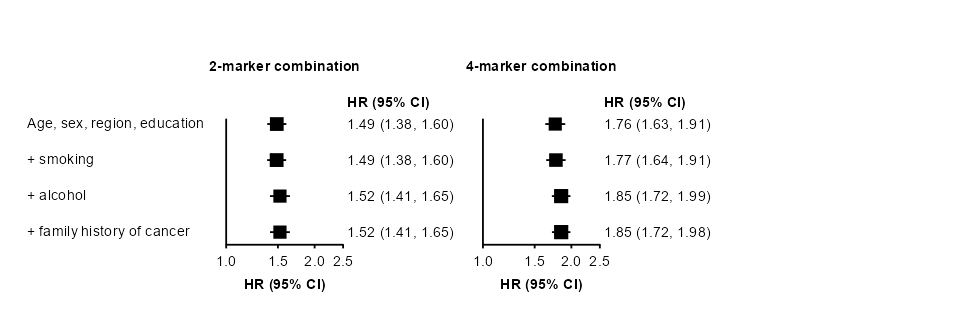
**

1. **Non-endemic areas (n=8 areas)**


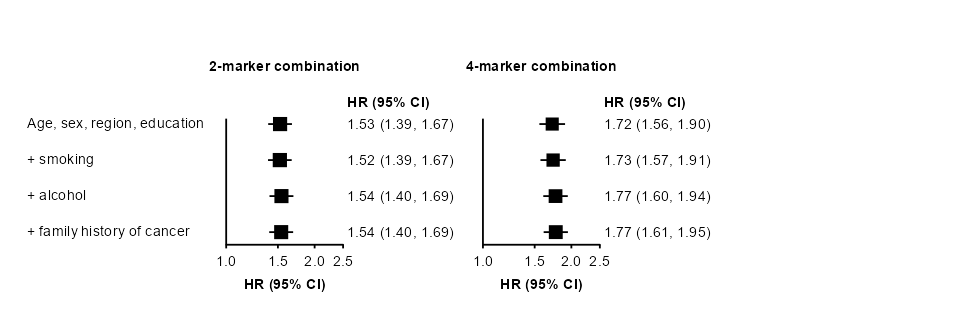


**(C) Endemic areas (n=2 areas)**


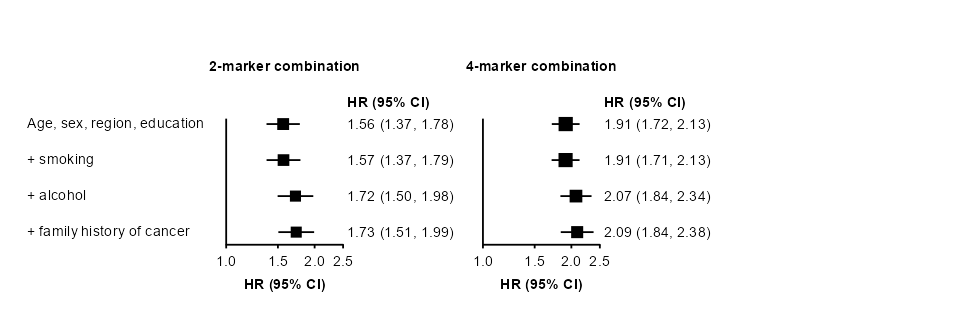


# **Figure S11. Distribution of MFI values of each EBV antigen among cases by the time interval between blood sample collection and cancer diagnosis**

The solid horizontal lines are the median MFI for each time group. The box covers the first and third quartile of MFI. The whiskers extend to the most extreme data point which is no more than 1.5 times the interquartile range from the box; Abbreviations: MFI - median fluorescence intensity; EBV - Epstein-Barr virus

**
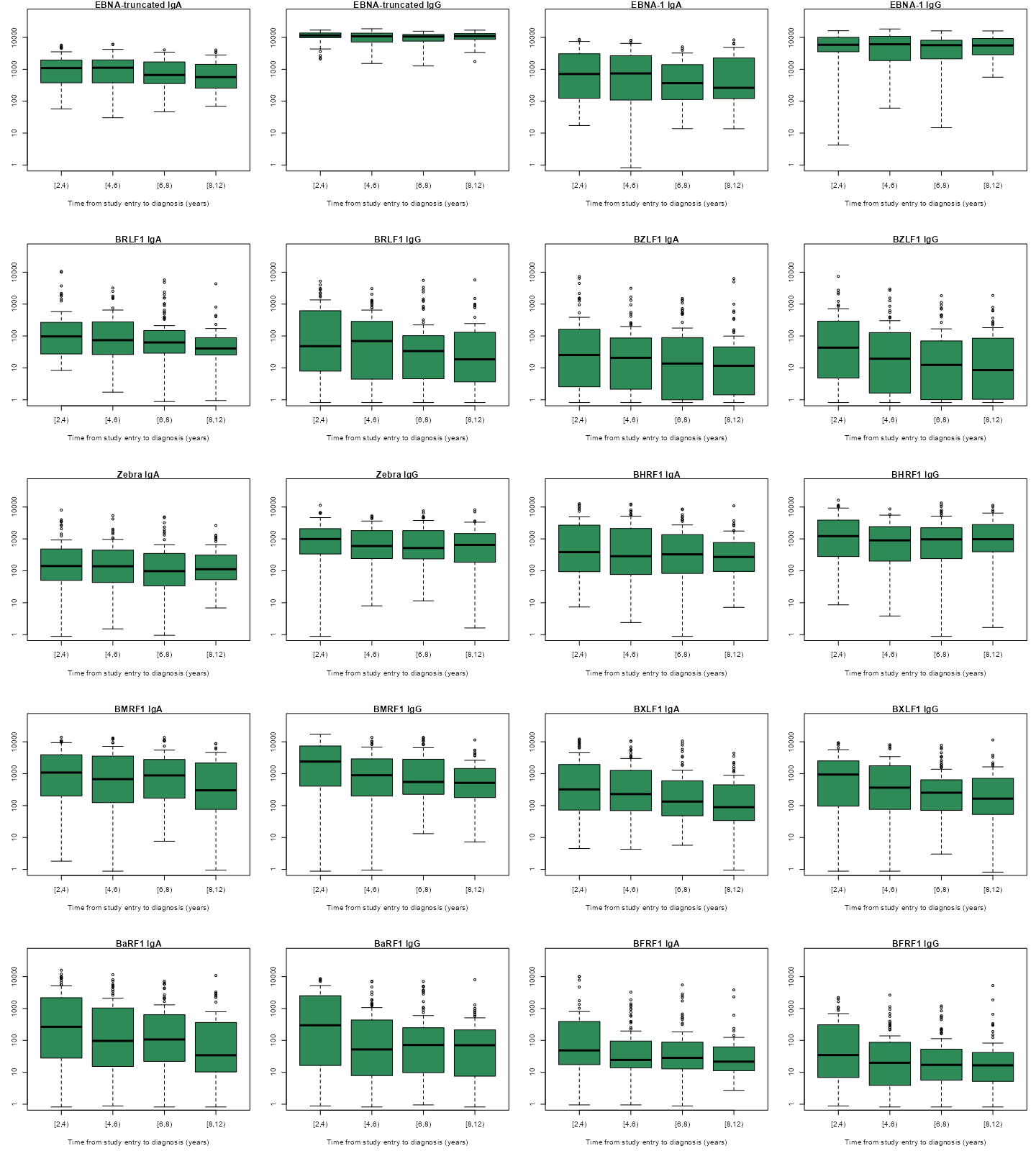
**

**
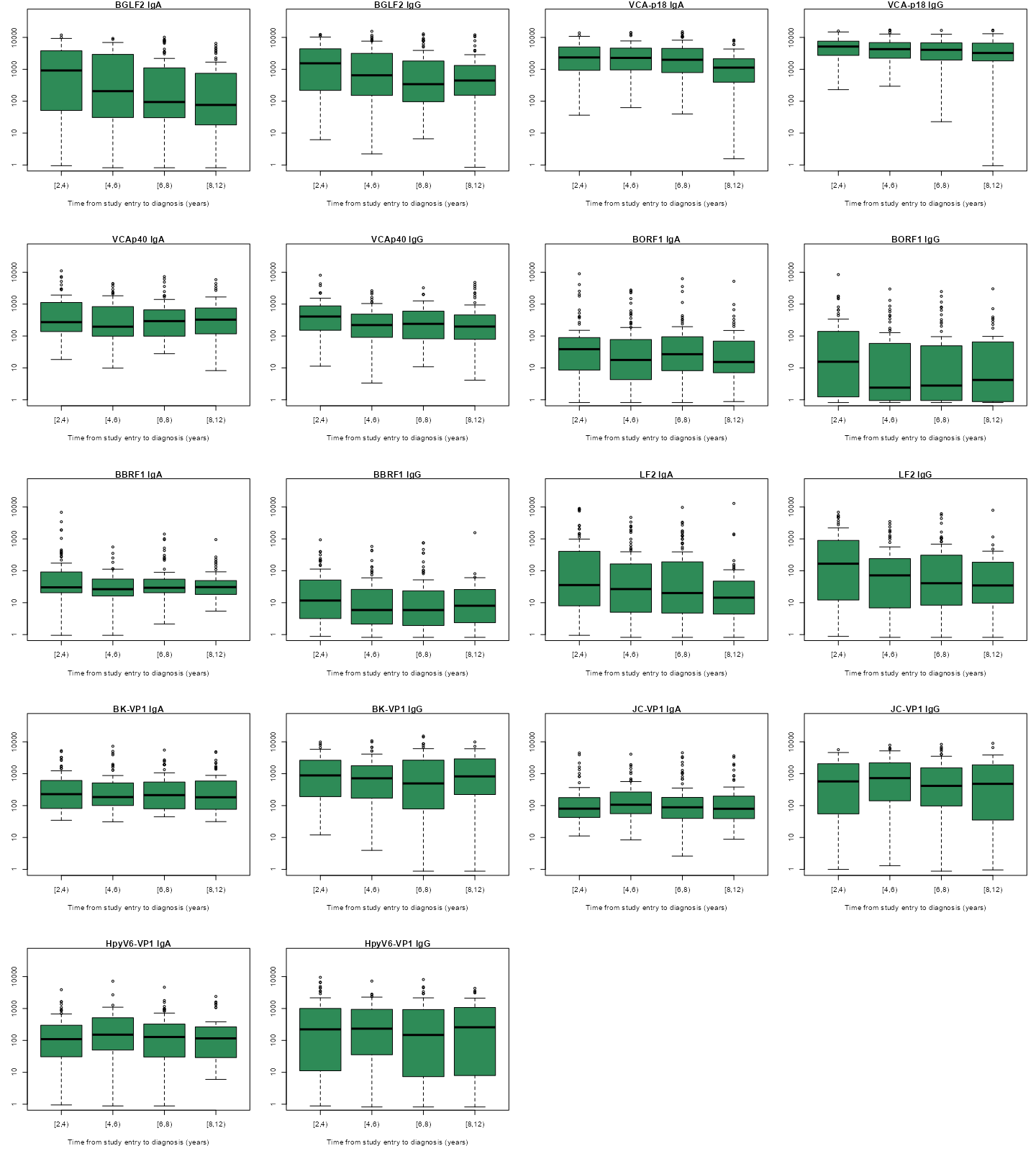
**
